# Controlling Microbial Spoilage of Aquatic Products with Hurdle Technology: A Review

**DOI:** 10.3390/foods15203621

**Published:** 2026-10-12

**Authors:** Meng Liu, Shuai Gao, Xinran Tao, Bingshuang Liu, Feifei Gong, Yang Yang, Yiyu Chen, Guangming Liu

**Affiliations:** 1 School of Marine Biology, Applied Technology Engineering Center of Fujian Provincial Higher Education for Marine Food Nutrition Safety and Advanced Processing, Xiamen Ocean Vocational College, Xiamen 361100, China; liumeng@xmoc.edu.cn (M.L.); gongfeifei@xmoc.edu.cn (F.G.); chenyiyu@xmoc.edu.cn (Y.C.); 2 College of Ocean Food and Biological Engineering, Jimei University, Xiamen 361021, China; 202414908041@jmu.edu.cn (S.G.); 15140524539@163.com (X.T.); liubingshuang2025@163.com (B.L.); 3 College of Environment and Public Health, Xiamen Huaxia University, 288 Tianma Road, Xiamen 361024, China; yangy@hxxy.edu.cn

**Keywords:** antimicrobial mechanisms, aquatic products, hurdle technology, intelligent packaging, preservation, spoilage microorganisms

## Abstract

Spoilage due to microbial proliferation is the primary cause of the decline in the quality and nutritional value of aquatic products. Current research largely focuses on various antimicrobial methods, yet each antibacterial preservation technology has certain limitations in maintaining storage quality and prolonging the shelf life of aquatic products. This paper categorizes the spoilage microorganisms of aquatic products and highlights that *Shewanella* spp. and *Pseudomonas* spp. are common spoilage organisms. The multiple antimicrobial mechanisms of hurdle technology are examined: combined hurdles are classified into five patterns (physical–physical, physical–chemical, physical–biological, biological–chemical, and biological–biological), and their aquatic applications are reviewed together with emerging intelligent controlled-release antibacterial packaging. Aims to provide theoretical references for the iterative optimization of aquatic product preservation technologies.

## 1. Introduction

Food spoilage poses a global challenge, exerting detrimental effects on food security, nutritional quality, and human health [1]. Owing to their delicate taste and high-nutrient contents such as high-quality protein, aquatic products are highly popular among consumers globally [2]. Their freshness is a critical factor that directly determines seafood sensory quality and nutritional value. Microorganisms attached to the surface of aquatic products and present in the surrounding environment proliferate rapidly under high-moisture and nutrient-rich conditions, rendering aquatic products highly prone to spoilage during harvesting, transportation, and storage [3]. This process not only deteriorates the quality and nutritional value of aquatic products but also gives rise to potential food safety risks, thereby posing a serious threat to consumer health.

Any factors that can inhibit the growth and reproduction of microorganisms in food are defined as hurdle factors [1]. Hurdle technology constructs multiple antibacterial barriers by integrating physical, chemical, and biological hurdle factors to restrain microbial proliferation and delay the spoilage of aquatic products [1]. Physical hurdle technologies mainly include low-temperature storage, pH regulation, water activity control (such as salt curing), modified atmosphere packaging (MAP), ultrasound, and other techniques [4,5]. Common chemical hurdle technologies refer to artificially added chemical preservatives and antioxidants, including sorbic acid, butylated hydroxytoluene, and butylated hydroxyanisole. Biological hurdle technologies are typically represented by microbial inhibitors (for instance, nisin and lysozyme), microbial antagonism, natural extracts (including organic acids, plant essential oils, and tea polyphenols), and quorum-sensing (QS) inhibitors [1,6,7,8]. Single-hurdle preservation technology can extend the shelf life of aquatic products to a certain extent, but it generally suffers from high application requirements and limited applicability [5]. Physical hurdle technologies are highly dependent on specialized instruments and suffer from relatively high application costs. Chemical hurdle techniques carry potential risks of chemical residues, and their safety performance requires further in-depth evaluation. For biological hurdle technologies, large-scale implementation and cost-effective control constitute the major bottlenecks restricting their widespread practical application. Combined hurdle technology exhibits prominent synergistic preservation effects [9]. Different hurdle factors act on microbial cell membrane structure, energy metabolism pathways, gene expression, and QS systems, forming multi-dimensional antibacterial targets and achieving an enhanced preservation effect [10,11].

The cited references were manually selected by the authors based on domain expertise to collect representative, high-quality publications closely related to aquatic product preservation, hurdle technology and spoilage microorganisms. This paper categorizes the common spoilage microorganisms in fish, shrimp, and shellfish, with emphasis on the dominant spoilage *Shewanella* spp. and *Pseudomonas* spp. It summarizes the multiple antimicrobial and preservation mechanisms of hurdle technology, classifies combined hurdle technologies into five combinatorial patterns (physical–physical, physical–chemical, physical–biological, biological–chemical, and biological–biological), discusses their applications in aquatic product preservation, and introduces emerging intelligent controlled-release antibacterial packaging. Furthermore, it outlines the future research priorities of combined hurdle technology. It is expected to provide a theoretical reference for scheme selection and iterative optimization of aquatic product preservation technologies.

## 2. Spoilage Microorganisms of Aquatic Products

Microorganisms are a major cause of aquatic product spoilage. The microorganisms that dominate the spoilage process are defined as specific spoilage organisms (SSOs) [12]. SSOs are important microbial groups that initiate the spoilage process of fresh aquatic products. The predominant SSOs mainly include *Pseudomonas*, *Shewanella*, *Vibrio*, *Bacillus*, *Photobacterium*, *Acinetobacter*, *Aeromonas*, *Lactobacillus*, and *Saccharomyces* [13]. Most of these organisms are Gram-negative. *Pseudomonas* spp., *Shewanella* spp., *Photobacterium phosphoreum*, and lactic-acid bacteria (*Lactobacillus*, *Carnobacterium*) act as major SSOs under corresponding storage conditions. Meanwhile, Vibrionaceae, Enterobacteriaceae, and *Bacillus* spp. include strains with notable pathogenic potential beyond their spoilage-related activities. Although these spoilage microorganisms account for a small proportion of the initial microbial community, they can rapidly proliferate under favorable conditions and become the dominant microbiota.

The composition of spoilage microbial communities in aquatic products is strongly dependent on environmental and storage conditions. The initial microbiota of fresh aquatic products is dominated by Gram-negative bacteria, within which fermentative members of the family *Vibrionaceae* are frequently among the dominant taxa [14]. Aquatic products harvested from polluted water are dominated by Enterobacteriaceae [15]. Under refrigerated conditions, psychrotolerant *Pseudomonas* spp. and *Shewanella* spp. often become the main spoilage bacteria of aquatic products, including cold-stored shrimp [16]. Under vacuum packaging (VP) or MAP, the growth of strictly aerobic Gram-negative spoilage bacteria (e.g., *Pseudomonas* spp. and *Shewanella* spp.) is inhibited. The spoilage microbiota shifts toward CO_2_-tolerant and facultatively anaerobic taxa, notably *Photobacterium phosphoreum* and lactic acid bacteria including *Lactobacillus* and *Carnobacterium* spp. [17,18]. In mildly processed aquatic products with low salt, low water activity, and slight acidification, the spoilage microbiota is more complex, often including lactic acid bacteria (such as *Lactobacillus* and *Carnobacterium*), as well as psychrotolerant Enterobacteriaceae and *Photobacterium* [13]. In semi-preserved products such as marinated or salted fish, acidification and low-salt conditions promote the growth of *Lactobacillus* and yeasts, thereby inhibiting the proliferation of other spoilage bacteria. In sous vide products subjected to mild heat treatment, the main SSOs are *Bacillus* or *Clostridium* [13]. Most bacteria attached to the surface of freshwater aquatic products are mesophilic and are easily inhibited under low-temperature storage, so their SSOs are usually psychrotrophic bacteria such as *Pseudomonas* and *Shewanella* [19,20,21].

The packaging method is also a key factor affecting the composition of SSOs. *Aeromonas* spp., *Acinetobacter* spp., *Brochothrix thermosphacta*, *Moraxella* spp., *Psychrobacter* spp. *Pseudomonas* spp. and *Shewanella* spp. are common aerobic SSOs in aquatic products. Under aerobic conditions, *Pseudomonas* and *Shewanella* often act as the dominant spoilage bacteria in aquatic products [3,6]. In contrast, under VP or MAP, changes in the gaseous environment inhibit aerobic spoilage bacteria and promote the growth of microorganisms adapted to low-oxygen or high-CO_2_ conditions, thereby altering the structure of the spoilage microbiota. Relevant studies have also confirmed that the main spoilage bacteria in refrigerated fish include *Pseudomonas*, *Shewanella putrefaciens*, *Aeromonas*, *Photobacterium*, and *Brochothrix thermosphacta*. Their dominance will undergo dynamic changes under processing and packaging interventions.

The composition of SSOs varies significantly among different aquatic products [22]. This variation is not only related to the species and living environment of the aquatic products, but also influenced by transportation, storage, and processing conditions [3]. Studies have shown that even within the same species, individuals may carry different initial microbial communities due to differences in living environment, handling, and storage conditions, leading to a distinct succession of dominant microbiota during spoilage [23,24]. However, under identical conditions, the same species usually develops a relatively stable spoilage microbiota.

**Figure 1:**
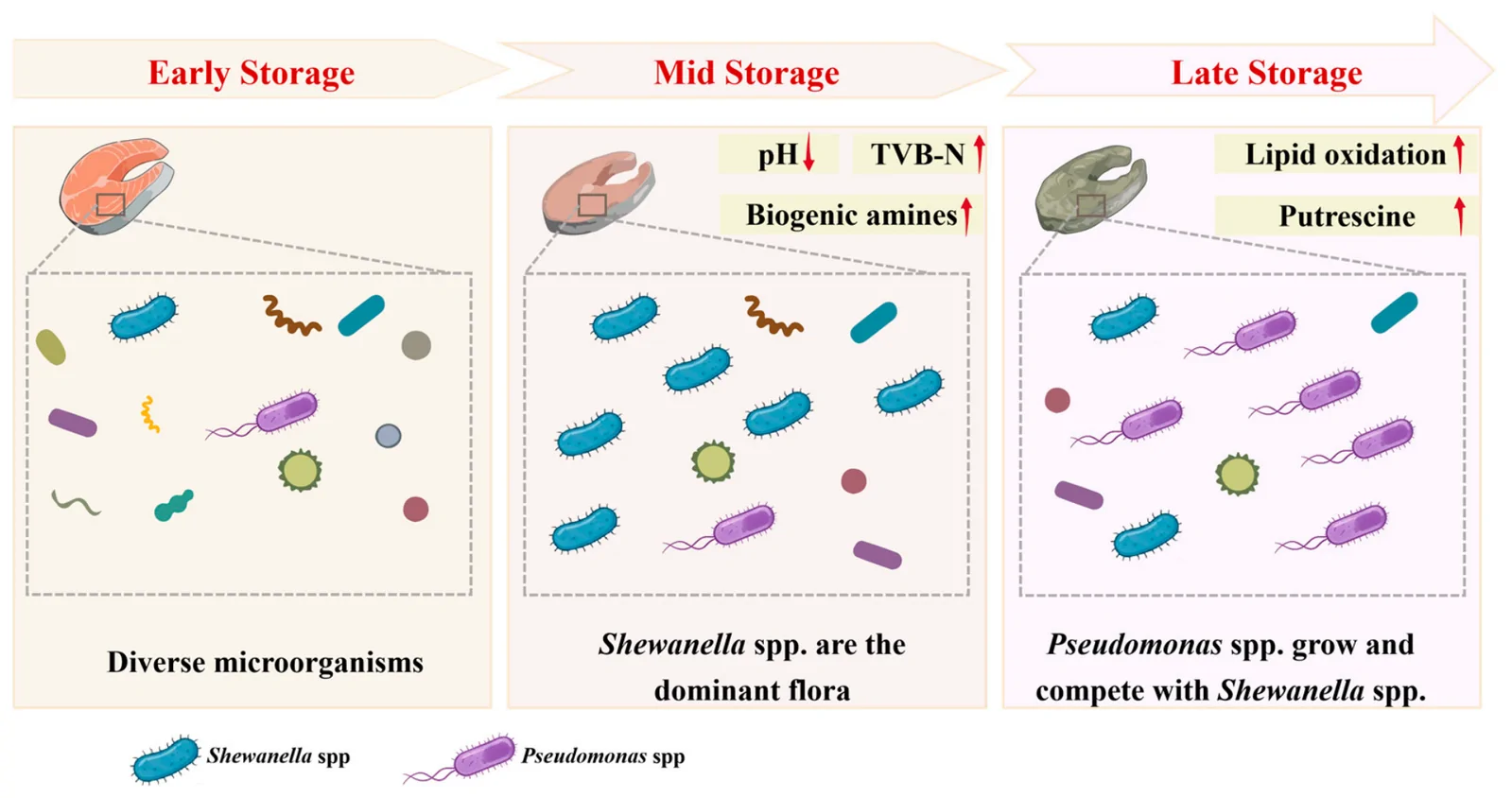
Schematic diagram of aquatic product spoilage coupled with microbial growth.

*Pseudomonas* spp. and *Shewanella* spp. are the common spoilage organisms in fish, including carp, grass carp, and silver carp [3,25,26], with particularly high abundance in deteriorated carp muscle [23]. *Pseudomonas fluorescens* serves as the predominant SSO in tuna and turbot [6]. *Pseudomonas putida* was isolated from grass carp fillets, and it was found that this strain possesses a strong capacity to assimilate and metabolize free amino acids, which accelerates the spoilage of grass carp [27]. *S. putrefaciens* is reported as the predominant spoilage bacterium of large yellow croaker [28]. As chilled storage proceeds, *Shewanella* spp. continuously carry out metabolic activities to reduce trimethylamine N-oxide to trimethylamine, which facilitates a competitive advantage over other microorganisms, enabling persistent proliferation and making them one of the dominant spoilage microbiota [29]. Subsequently, biogenic amines, such as putrescine, accumulate progressively, accompanied by a continuous increase in total volatile basic nitrogen (TVB-N) levels, leading to gradual softening and off-odor development in aquatic products. Under low-temperature conditions, *Pseudomonas* spp. promote the formation of lipid oxidation products, exacerbate off-flavor generation, and compete with *Shewanella* spp. for iron ions. As *Pseudomonas* spp. grow, their metabolites increase putrescine production, resulting in a pronounced sour and putrid odor, mushy muscle texture, gray-green discoloration on the surface, and significant quality deterioration of aquatic products (Figure 1).

The spoilage process of shrimp is affected by multiple factors, such as species and storage environments, and a wide variety of spoilage bacteria can be detected in shrimp. *Pseudomonas*, *Vibrio*, and *Shewanella* are common dominant spoilage bacteria [3,6]. The bacterial microbiota associated with fresh Indian white shrimp mainly includes *Aeromonas*, *Pseudomonas*, *Vibrio*, *Flavobacterium*, and *Serratia* [30]. The SSOs in *Penaeus vannamei* (syn *Litopenaeus vannamei*) belonged to *Shewanella*, *Psychrobacter*, *Pseudomonas*, *Acinetobacter*, and *Vibrio* [16]. The SSOs of *Litopenaeus vannamei* (syn *Penaeus vannamei*) were identified as *Psychrobacter*, *Shewanella*, and *Flavobacterium* using proteomics and 16S rRNA sequencing [31]. During storage at 25 °C, *Vibrio* dominated the microbiota of Pacific white shrimp. At 4 °C, the dominant genera shifted to *Acinetobacter*, *Psychrobacter*, and *Shewanella* [32]. During storage at 4 °C, the dominant spoilage bacteria in crayfish tails successively shifted from *Streptococcus* to *Pseudomonas*, and finally to *Paenisporosarcina* [33].

*Shewanella* is also a major spoilage bacterium in shellfish under refrigeration. For refrigerated Pacific oysters, the dominant spoilage bacteria are *Pseudoalteromonas* and *Shewanella*, while *Psychrobacter* becomes predominant after high-pressure treatment [34]. In refrigerated blue mussels, *P. arcticum* and *S. frigidimarina* serve as the dominant microbes [35].

During the processing and storage of aquatic products, microbial growth and metabolism, as well as endogenous enzyme activity, are closely related to the edible quality of aquatic products [5]. Identifying typical spoilage bacteria of various aquatic products and precisely inhibiting the proliferation of SSOs are essential for stabilizing product quality and extending shelf life [9].

## 3. Antimicrobial Mechanisms of Hurdle Technology

Microbial growth and reproduction constitute a major cause of aquatic product spoilage. The antimicrobial-preservation mechanism of hurdle technology mainly involves disrupting the structural integrity of microbial cells, interfering with microbial energy metabolism, regulating microbial gene expression, altering microbial living microenvironments, and inhibiting the QS system.

### 3.1. Disruption of Microbial Structural Integrity

Disrupting structural integrity constitutes the foundation of most antimicrobial mechanisms. Physical and chemical treatments directly damage the outer barriers of microbial cells, disrupt intracellular homeostasis, and eventually lead to microbial inactivation. High pressure, such as high hydrostatic pressure (200–500 MPa), killed histamine-forming bacteria by first-order kinetics; it caused cell-wall blistering, swelling, rupture and cytoplasmic leakage, indicating the cell envelope as the primary damage site [36]. Ozone destroys membrane proteins and lipopolysaccharides via strong oxidation, triggering the leakage of intracellular contents [37]. Pulsed electric fields and pulsed light disrupt membrane integrity, increase permeability, and eventually inactivate microbial cells [38]. Slightly acidic electrolyzed water can also impair bacterial cell walls and disrupt cell membrane permeability [39]. Phenols, alcohols, and other active components in plant essential oils are capable of penetrating bacterial cell membranes, destroying membrane structures, and causing the release of intracellular substances [22]. As a cationic polysaccharide, chitosan adheres to the negatively charged surface of bacterial cell membranes through electrostatic interaction, increases membrane permeability, induces the outflow of intracellular contents, and ultimately inhibits microbial growth [40].

Nevertheless, bactericidal efficacy depends on treatment parameters, bacterial physiological state, and interference from the food matrix. Protein- and lipid-rich aquatic matrices can shield microbial cells, so inactivation in real food systems is often weaker than that measured in pure culture. Physical treatments applied at sufficient intensity generally cause irreversible lethal damage, whereas chemical and biological treatments at practical doses tend to be bacteriostatic rather than bactericidal. In addition, the outer membrane of Gram-negative spoilage bacteria such as *Shewanella* spp. and *Pseudomonas* spp. limits their susceptibility to hydrophobic plant extracts and chitosan.

### 3.2. Inhibition of Microbial Energy Metabolism

Microbial physiological processes require steady energy supplementation. Suppression of energy metabolic pathways can efficiently hinder microbial growth and proliferation.

Multiple preservatives are capable of suppressing the activity of vital enzymes in microorganisms. Slightly acidic electrolyzed water has been verified to inactivate endogenous enzymes in aquatic products, including polyphenol oxidase, lysosomal enzymes, and cathepsins [39]. Plant essential oils and polyphenolic compounds exert inhibitory effects on bacterial enzymes, including ATPases and protein synthases, thereby disrupting microbial metabolic homeostasis [41]. In seafood species, trimethylamine-N-oxide reductase (TorA), a distinctive enzyme derived from *Shewanella*, is the pivotal catalyst for the generation of fishy off-flavor. The suppression of TorA activity is conducive to inhibiting trimethylamine biosynthesis at the source [15].

Aldehydes constrain microbial proliferation by perturbing intracellular energy metabolism networks. Certain aldehyde derivatives, such as (*E*)-2-hexenal [42] and cinnamaldehyde [43], trigger excessive accumulation of reactive oxygen species (ROS), which impairs cytoplasmic membrane integrity, disturbs glycolytic pathways and ATP biosynthesis, and ultimately exerts potent inhibitory effects on microbial growth [44].

Nevertheless, inhibiting metabolism and enzyme targets usually leads to inhibition of bacterial growth rather than complete eradication of the bacteria. As preservative concentrations decline within complex aquatic food matrices, inhibited cells may restore enzyme activity and energy metabolism and resume growth. Notably, TorA inhibition is confined to *Shewanella* and cannot block off-flavour formation arising from spoilage metabolites produced by other SSOs.

### 3.3. Regulation of Microbial Gene Expression

Intervention in microbial gene expression can suppress the synthesis of virulence factors, metabolic products, and essential proteins at the molecular level, thereby restraining microbial growth and proliferation.

Beyond direct macromolecular damage, irradiation and photodynamic inactivation regulated bacterial gene expression. A 0.4 kGy γ-irradiation dose triggers a global stress response in *Escherichia coli* O157:H7 that recruits the SOS and excision–repair regulons, oxidative-stress and heat-shock genes, and simultaneously induces virulence determinants such as Shiga toxin and type III secretion genes [45]. Photodynamic inactivation remodels the transcriptome in a comparably broad, ROS-centred manner, typically downregulating quorum-sensing and virulence genes.

The expression of toxigenic and metabolic genes can be directly repressed via binding to DNA promoter regions or interfering with transcription factor activities. For example, cuminaldehyde binds to DNA through hydrogen bonding, loosening the DNA double-helix structure and inhibiting the proliferation of *Staphylococcus aureus* [46]. Whereas citral suppresses aflatoxin B_1_ biosynthesis by down-regulating aflR and the associated structural genes at sub-inhibitory concentrations [47], perillaldehyde acts mainly by inducing excessive ROS accumulation [48].

### 3.4. Modulation of Microbial Microenvironment

Regulation of storage conditions, such as temperature, pH, and water activity, for aquatic products can directly or indirectly inhibit microbial growth [2].

Low-temperature conditions, such as refrigeration and freezing retard aquatic product spoilage by reducing microbial metabolic rates and endogenous enzymatic activity [2,49], while ultra-low temperature exerts stronger suppression on microbial proliferation. Acidification strategies, including organic acid addition and CO_2_-MAP [50], reduce the surface pH of aquatic products and establish an acidic microenvironment unfavorable for the proliferation of most spoilage bacteria. High CO_2_-MAP can markedly inhibit melanosis development and microbial proliferation in Pacific white shrimp [15]. Reduction in water activity by means of drying, curing, and salting constrains the available water for microorganisms, thereby repressing their growth and metabolic processes [9].

### 3.5. Interference with Microbial QS System

Food spoilage microbes tend to colonize compact multispecies biofilms, whose formation and maturation are governed by QS systems [51]. QS enables bacteria to coordinate collective behaviors via signaling molecules. Disruption of this regulatory system can effectively weaken bacterial pathogenicity and spoilage potential.

Signal molecule synthesis can be suppressed by inactivating synthases or depleting corresponding substrates. For example, zingerone hinders the QS system of *Pseudomonas aeruginosa* via inhibiting *N*-acyl-L-homoserine lactone synthase activity [52]. Additionally, the quorum-sensing inhibitor resveratrol suppressed gene sb1370 expression and thereby attenuated QS and spoilage of fish fillets [53].

Spoilage and pathogenic bacteria enhance environmental stress resistance via biofilm formation. QS inhibitors can dismantle biofilm structures and attenuate microbial viability and pathogenicity. As natural bioactive compounds, furanocoumarins exert prominent inhibitory effects on biofilm formation of *Salmonella typhimurium*, *E. coli*, and *P. aeruginosa* [54].

According to the action modes of hurdle factors such as temperature, oxygen, and water activity, hurdle technologies are mainly divided into physical, chemical, and biological preservation technologies [8,55]. Table 1 outlines the differences among common hurdle technologies. Physical preservation technologies mainly consist of low-temperature storage [56], MAP [15], ozone treatment [37], and irradiation [57,58]. Although these methods have been widely utilized, they rely heavily on specialized equipment and substantially raise energy consumption during production. Low-temperature preservation requires strict temperature control. Slight temperature fluctuations may deteriorate product quality, which increases production costs and leads to economic losses. Chemical preservation approaches primarily rely on antimicrobial preservatives (e.g., sorbic acid and its salts, benzoates, sulfites and lactates) and on synthetic antioxidants (e.g., butylated hydroxytoluene and butylated hydroxyanisole), the former inhibiting microbial growth and the latter retarding lipid oxidation and rancidity [59,60]. Consumer concern over the safety of chemical synthetic preservatives, together with a stated preference for natural alternatives, has been a major driver for replacing chemical preservatives in aquatic product preservation [50]. Biological preservation technology refers to the preservation of products using biological preservatives. It can be divided into antimicrobial agents, antioxidants, enzyme preparations, and other types [61]. Among these, bacteriostats and enzyme preparations are currently the most extensively studied [3,62,63]. Bacteriocins are ribosomally synthesized antimicrobial peptides produced by a broad spectrum of bacteria that can inhibit foodborne pathogens and spoilage bacteria. Nisin and pediocin primarily target Gram-positive species via binding to lipid II, which disrupts membrane integrity and cell-wall biosynthesis [64]. Antibacterial peptides, which exhibit broad-spectrum inhibitory activity against microorganisms including *Aeromonas hydrophila*, *P. fluorescens* and *Pseudomonas stutzeri*, have also been identified and characterized [65,66,67]. Nevertheless, the practical application of such biopreservatives is generally restricted by high production costs, poor stability, and the difficulty in accurately evaluating and predicting their preservation efficacy.

Microbial growth and reproduction are regulated by multiple factors. Single hurdle technology exhibits limited antibacterial efficiency, along with notable drawbacks such as nutrient loss and quality deterioration of aquatic products [39]. Combined hurdle technology inhibits the metabolism and proliferation of spoilage microorganisms via multi-factor synergistic effects at multiple targets, delivering a more prominent preservation effect, and has become a mainstream research direction in the field of aquatic product preservation.

## 4. Application of Combined Hurdle Technology

Common combined hurdle technologies include physical–physical, physical–chemical, physical–biological, chemical–biological, and bio–biological hurdle technologies. These combinations have been widely investigated for the preservation of fish, shrimp, and shellfish, and can effectively extend their shelf life.

### 4.1. Physical–Physical Approaches

Physical preservation technologies mainly extend the shelf life of aquatic products by adopting physical means and specialized equipment without adding chemical substances or altering inherent food properties. In addition to low-temperature treatments such as refrigeration and partial freezing, emerging physical technologies including electrostatic fields, ultra-high pressure, and magnetic fields have been gradually applied to the preservation of aquatic products. Low-temperature treatment can effectively inhibit endogenous enzyme activity and microbial growth and is thus widely applied in the preservation of aquatic products. Nevertheless, it carries potential risks such as ice crystal formation and the subsequent decline in the sensory quality of products. The electrostatic field can induce the formation of fine and uniform ice crystals inside aquatic products, improve flesh color and sensory properties, and reduce drip loss. The electrostatic field mainly exerts auxiliary regulatory effects on ice-crystal morphology, water distribution and protein stability, with weak direct antimicrobial capacity. The combined treatment of a low-voltage electrostatic field and partial freezing can effectively reduce the relative abundance of spoilage bacteria such as *Pseudomonas* and *Psychrobacter* in large yellow croaker, decrease TVB-N and total viable count values, delay spoilage, and extend the shelf life [11]. Ultra-high pressure technology inactivates microorganisms and induces protein denaturation in aquatic products, thereby efficiently prolonging shelf life. However, it is highly parameter-dependent, and inappropriate pressure may trigger undesirable protein denaturation. The magnetic field regulates water distribution in frozen aquatic products, modulates protein hydration and conformational changes, and consequently retards quality deterioration, though it has a weak direct antimicrobial capacity. The combined treatment of 300 MPa ultra-high pressure and magnetic field can inhibit lipid oxidation and protein denaturation, suppress microbial growth, and extend the shelf life of grass carp fillets [10].

### 4.2. Physical–Chemical Approaches

Chemical preservation technologies exert bactericidal effects by the exogenous addition of antimicrobial substances, which interfere with and disrupt physicochemical reactions and metabolic activities within microbial cells, ultimately leading to microbial death. Chemical preservatives, represented by slightly acidic electrolyzed water, have gradually become a research hotspot in aquatic product preservation. Hypochlorous acid in slightly acidic electrolyzed water targets the cell wall, cell membrane, and intracellular components of microorganisms to achieve bactericidal efficacy and effectively reduce common foodborne pathogens. However, its antimicrobial performance is closely related to pH conditions, and long-term application may cause surface corrosion and damage to processing equipment [39]. The combined treatment of slightly acidic electrolyzed water and freezing significantly suppressed myofibrillar and sarcoplasmic protein degradation in *L. vannamei*. At day 7 of storage, it achieved 71.6%, 47.8%, and 100% inhibition against spoilage bacteria such as *Psychrobacter*, *Shewanella*, and *Flavobacterium*, respectively, and extended shrimp shelf-life by disrupting the association between protein changes and spoilage bacterial growth [31].

### 4.3. Physical–Biological Approaches

QS inhibitors contribute to biofilm disassembly and attenuate microbial viability and virulence. As QS inhibitors, electrospun fibers prepared by coaxial electrospinning disturb microbial QS systems, repress *P. fluorescens* growth and the formation of TVB-N and trimethylamine, delay muscle tissue deterioration, and prolong the shelf-life of refrigerated salmon fillets [72]. Based on their sources, biological preservatives can be classified into four categories: plant-derived, animal-derived, microbial-derived, and enzyme-based preservatives. Plant polyphenols have been widely applied in the preservation of aquatic products. Their antimicrobial mechanisms involve disrupting the structure of bacterial cell walls and membranes, inhibiting biofilm formation, interfering with enzyme activity and protein regulation, as well as chelating essential metal ions for bacterial growth [5]. Phenolic extracts derived from grape seeds, lotus receptacles, and lotus roots, in combination with 4 °C cold storage, can reduce the relative abundance of Proteobacteria and reshape the microbial community structure of fish flesh, respectively. Such composite treatment significantly decreases TVB-N content, inhibits lipid oxidation, improves edible quality, and prolongs the shelf life of refrigerated fish meat [73]. Chitosan serves as an important broad-spectrum animal-derived biological preservative by disrupting bacterial cell walls and regulating microbial metabolism [5]. For grass carp stored at 4 °C, chitosan emulsion embedded with peptide MOp2 from moringa seeds suppressed proliferation of *E. coli* and *S. aureus*. The treatment brought down pH and mitigated TVB-N accumulation, prolonging the product shelf-life to 9 days [74]. Bacteriocins, as natural antimicrobial agents of microbial origin, can form a protective barrier on the surface of aquatic products to prevent the invasion of potential spoilage microorganisms. For sardines stored at 7 °C, *Lactiplantibacillus plantarum* OV50 not only persistently represses the growth of multiple food-borne pathogens (*P. aeruginosa* ATCC 27835, *S. aureus*, *E. coli* ATCC 8739, *Vibrio cholerae* ATCC 14035), but also lowers the abundances of Enterobacterales, coliforms, *Pseudomonas*, *Vibrio* and *S. aureus*. It further alleviates TVB-N accumulation and extends sardine shelf-life to 12 days [75]. Enzymes exhibit excellent antibacterial and antifungal activities, and thus can inhibit the growth of spoilage microorganisms responsible for aquatic product deterioration and prevent food spoilage [3]. Under −3 °C superchilled storage, a lysozyme-gum tragacanth-sodium alginate active coating suppresses the formation of TVB-N, malondialdehyde, histidine (a taste-active amino acid), and fishy off-flavor compounds in large yellow croaker (*Larimichthys crocea*). This treatment also mitigates water migration, and desirable sensory attributes can be retained in large yellow croaker following 7-day storage [76].

### 4.4. Biological–Chemical Approaches

Edible or biodegradable films and coatings incorporated with biological preservatives can also be applied to extend the shelf life of aquatic products. The synergistic preservation mechanism relies on the antibacterial efficacy of diverse biological preservatives, while the films and coatings prevent protein denaturation and water loss and form a protective barrier. This strategy maintains the freshness of aquatic products without altering their original flavor and texture properties [22,61]. Common edible films and coatings are primarily fabricated from biomacromolecules, including polysaccharides, proteins, and lipids [61]. Polysaccharide-based films exhibit excellent gas barrier properties, while protein-based films are renowned for their superior mechanical performance. However, films derived from polysaccharides and proteins generally show poor water vapor barrier ability [22].

Polysaccharide film matrices such as chitosan and cellulose extend shelf life mainly by inhibiting water migration and retarding oxidative deterioration. Composite chitosan films incorporated with gallic acid mitigate TVB-N build-up in Atlantic horse mackerel (*Trachurus trachurus*). By retarding microbial propagation and lipid peroxidation, the coating retains product eating quality and achieves prominent shelf-life extension [7]. Protein-based films and coatings can readily adhere to the surface of food matrices due to their hydrophilic characteristics, exerting a favorable effect on inhibiting water migration and further improving product added value. The incorporation of essential oils can further enhance the antimicrobial performance of protein-based coatings [61]. Zein-based composite coatings incorporated with plant essential oils bring down total microbial populations (Enterobacteriaceae, psychrotrophs, mesophiles and *Pseudomonas*) in rainbow trout muscle. By suppressing the rise in TVB-N and pH, this treatment retains favorable sensory properties of odor and color, achieving an effective shelf-life extension for rainbow trout [77]. Loading bio-preservatives into film matrices is a double-edged strategy. High doses of plant essential oils, polyphenols, or propolis extracts strengthen antimicrobial and antioxidant capacity, yet the same doses may weaken the mechanical integrity of the film and impart undesirable off-flavours to the aquatic product. In the near-saturated humidity of chilled aquatic storage, moisture sorption plasticises these films, lowering tensile strength and glass-transition temperature, raising water-vapour permeability, and partially dissolving water-soluble fractions, so the physical barrier weakens progressively. The incorporation of lipid fractions can ameliorate the limitations of polysaccharide- and protein-based films [22]. Lipids can increase film thickness, hinder water migration, and enhance the cohesiveness of edible coatings. The composite coating prepared by incorporating propolis extract into a chitosan film matrix can remarkably improve the sensory attributes of golden threadfin bream fillets, retard lipid oxidation, reduce the total viable counts of mesophilic bacteria, and consequently extend the shelf life of *Nemipterus japonicus* fillets [78].

### 4.5. Bio–Biological Approaches

Biological preservatives are mostly applied in compound formulations, which reduce the dosage of a single preservative and synergistically enhance antimicrobial and fresh-keeping performance [3]. Such synergy among biological preservatives arises from complementary action modes: one agent disrupts the cell envelope, facilitating entry of the second, so that membrane integrity, energy metabolism, or virulence-related gene expression is jointly disturbed. Nisin exerts broad-spectrum antibacterial activity against Gram-positive bacteria by altering cell membrane permeability. Phenolic compounds possess strong hydrophobicity and can trigger the leakage of intracellular substances. Their combination can effectively lower application dosage and greatly strengthen the inhibitory activity against *Listeria* [79]. It should be noted that such synergistic effects have been primarily demonstrated against *Listeria*. Since the outer membrane of Gram-negative bacteria prevents nisin from accessing lipid II, nisin exhibits markedly weaker activity toward *Shewanella* and *Pseudomonas*. Its inhibitory potency can be enhanced only after outer-membrane permeabilization induced by chelators such as EDTA. Consequently, aquatic-food applications of nisin depend on such synergistic partners. Representative examples include chitosan applied against *Shewanella* spp. isolated from large yellow croaker [80]. Typical aquatic spoilage organisms, including *Shewanella* spp. and *Pseudomonas* spp., are Gram-negative bacteria shielded by an outer membrane. Nisin is inherently weakly active against Gram-negative strains; it may even bind their anionic surface and stabilize the outer membrane rather than reach the cytoplasmic membrane.

Chitosan is commonly applied in film form to isolate air, thereby preventing microbial contamination and food oxidation [40]. ε-polylysine can effectively alleviate protein and lipid oxidation and maintain product quality. For stored Chinese shrimp, chitosan coatings incorporated with ε-polylysine and carrageenan deliver prominent antimicrobial effects. This composite treatment retards lipid oxidation and restrains the buildup of TVB-N, thereby optimizing shrimp quality and achieving a notable extension of shelf-life [81].

Synergy depends strongly on mixing ratio, total concentration, food–matrix interference, and storage temperature. Protein- and lipid-rich matrices may bind or partition active molecules and weaken practical efficacy.

Table 2 summarizes the reported applications of combined hurdle technology in aquatic product preservation. Current studies are mostly focused on the inhibition of spoilage bacteria such as *Pseudomonas* spp. and *Shewanella* spp. in fish, while *Shewanella* spp. are one of the common spoilage organisms for shrimp. *Shewanella* spp. and *Pseudomonas* spp. are both Gram-negative bacteria, yet they differ significantly in their tolerance to low temperatures. Compared with *Shewanella* spp., *Pseudomonas* spp. in aquatic products are usually more difficult to inhibit and require more sophisticated hurdle technologies. This phenomenon may be attributed to the fact that *Pseudomonas* spp. possess a complete Luxl/R-type QS system, a full type VI secretion system and a far richer pyoverdine-based iron-acquisition repertoire, all of which are largely absent in *Shewanella*. Existing studies have also demonstrated that the presence of *Pseudomonas* spp. can promote the growth of other spoilage microorganisms within mixed-species biofilms [51]. As a result, *Pseudomonas* outcompetes *Shewanella* in mixed-species biofilms at low temperature by repressing its metabolism, and AHL-mediated QS, together with the EPS matrix, raises the resistance of the community as a whole to disinfectants and other stresses [82]. Compared with single hurdle technology, which has limitations including the risk of inducing microbial stress tolerance, optimally formulated combined hurdle technology can maintain favorable physicochemical and sensory quality of products over a long storage period.

## 5. Future Perspectives

While abundant progress has been achieved regarding hurdle technology and antimicrobial strategies for aquatic products preservation, multiple critical knowledge gaps remain to be addressed for industry application. Below we summarize key findings from current studies, followed by explicit identification of unresolved challenges and prospective research priorities.

The proliferation of aquatic spoilage microorganisms, including typical spoilage organisms, is profoundly modulated by environmental factors such as temperature and oxygen. Accordingly, for aquatic products subjected to identical processing and storage conditions, the selection and combination of suitable hurdle technologies follow inherent regularities to realize targeted antibacterial and preservation effects [83]. The core task of future research lies in quantifying these regularities, rather than deriving them empirically from individual case-specific experiments.

*Shewanella* features strong low-temperature tolerance and high spoilage potential, serving as a typical spoilage microorganism in refrigerated aquatic products [19]. Its facultative anaerobic characteristic further increases the difficulty of preservation. The growth rate of *Shewanella* and other spoilage bacteria decreases significantly with the increase in carbon dioxide concentration [84]. MAP with optimized gas composition can effectively extend the shelf life of aquatic products [85]. For mariculture fish, including large yellow croaker, *Shewanella* and *Vibrio* are the dominant spoilage organisms. Accordingly, a combined hurdle system combining low temperature and high CO_2_-MAP is recommended [86]. *Pseudomonas* spp., common aerobic spoilage bacteria in aquatic products, biofilm formation and the production of extracellular matrix provide a further layer of protection against low temperature and other stresses [6,51]. As a natural bioactive substance, chitosan can damage the microstructure of bacterial cells and disrupt biofilms, thereby inducing the lysis and death of *P. aeruginosa* [87]. Freshwater fish, including carp and grass carp, are easily colonized by *Pseudomonas*, *Acinetobacter*, and *Aeromonas* during storage. Therefore, a combined preservation strategy integrating VP or MAP with biological preservatives such as chitosan is highly applicable. In addition, shrimp such as *L. vannamei* and Indian white shrimp are prone to melanosis caused by microbial proliferation and oxidation. Edible films and coatings serve as an effective covering strategy to enhance the barrier capacity to water vapor, O_2_, and CO_2_ and prolong shelf life [22,88]. The combination of edible coatings loaded with essential oils and other active substances with hurdle technologies, including low temperature and MAP, can exert synergistic preservation effects. This composite strategy integrates the physical barrier function of edible films, the antibacterial and antioxidant activity of plant-derived active components, and the metabolic inhibition effect of low temperature and MAP. It can markedly inhibit the growth and biofilm formation of typical aquatic spoilage bacteria, reduce the accumulation of TVB-N and other spoilage metabolites, and slow down lipid oxidation and texture deterioration [61].

**Figure 2:**
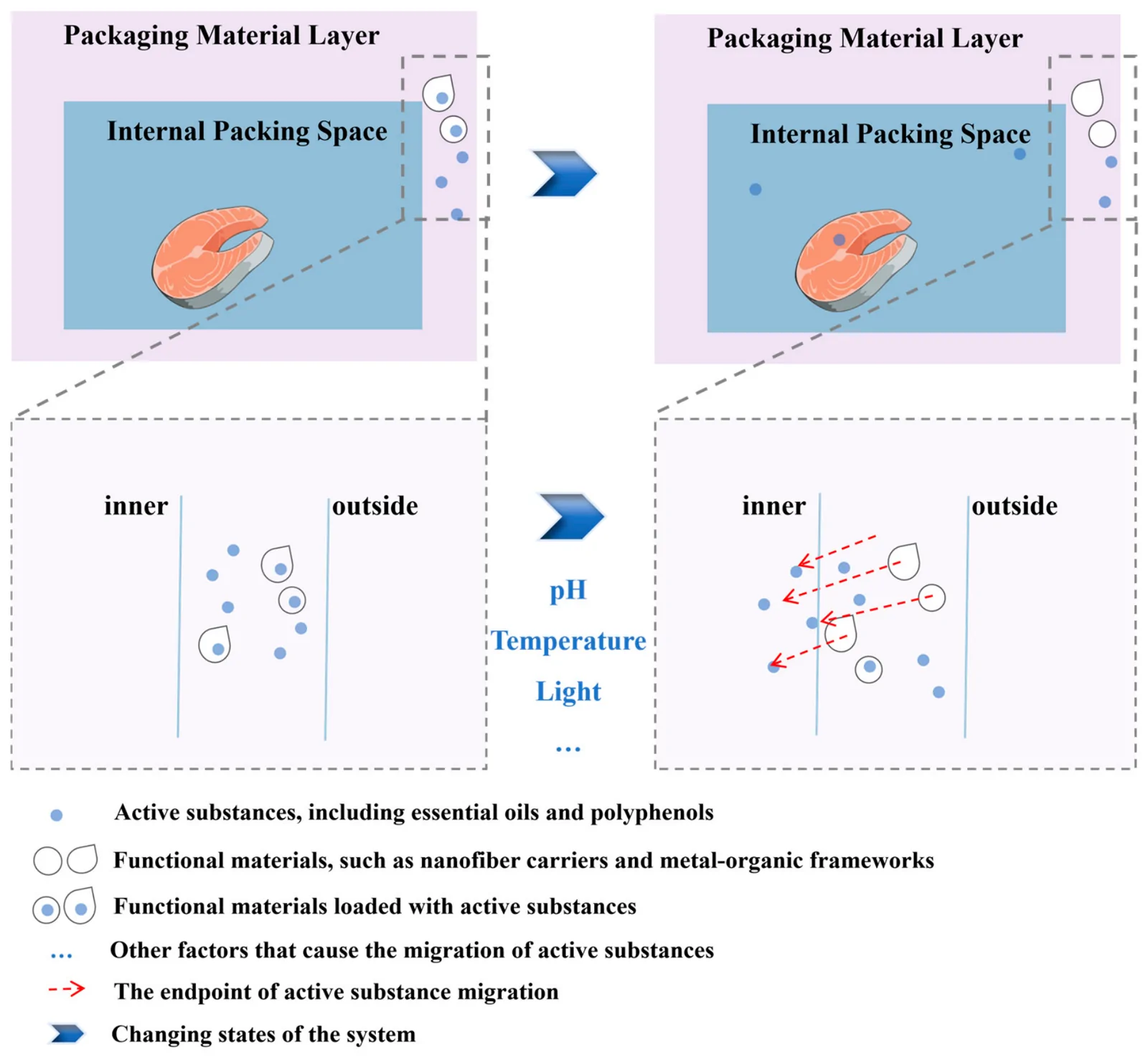
Schematic view of the release mechanism of intelligent controlled-release packaging.

Aquatic products contain rich high-quality protein, unsaturated fatty acids, and abundant trace nutrients, accompanied by high water activity and a weak endogenous antioxidant system. These inherent physicochemical properties render aquatic products extremely vulnerable to microbial colonization and metabolic spoilage during storage and circulation [89]. Rapid proliferation of dominant spoilage microorganisms, continuous lipid peroxidation, and uncontrolled endogenous enzymatic reactions are the three core driving factors responsible for sensory deterioration, nutritional loss, and significant shelf-life shortening of fresh aquatic products [3,22]. Intelligent packaging loaded with antibacterial active substances can sense internal and external environmental changes in real-time through physicochemical variations in packaging substrates, such as shrinkage, swelling, dissolution, functional group structural transformation, and molecular self-assembly [90]. This system enables precise and controllable regulation of the release rate of functional active preservatives, so as to maintain food quality and safety (Figure 2).

Common response modes of intelligent controlled-release packaging mainly include temperature-responsive, pH-responsive, light-responsive, humidity-responsive, and enzyme-responsive types. Temperature-sensitive polyurethane films loaded with antimicrobial agents such as carvacrol and cinnamaldehyde. These films alter their phase state and free volume in response to ambient-temperature shifts: soft-segment phase transition drastically raises film porosity from 19.81% at 25 °C to 33.67% at 35 °C, and from 48.49% at 55 °C to 68.24% at 65 °C [91]. Such pore-size variation changes film air permeability and triggers antimicrobial release; carvacrol exhibits a temperature-dependent linear-rising release profile that obeys first-order kinetics [92]. In contrast, pH-responsive systems rely on distinct triggering mechanisms. Antimicrobial hydrogels fabricated from oxidized nanocellulose and nisin through electrostatic interactions can be integrated with paper-based packaging. Once food-system pH surpasses the isoelectric point of nisin, weakened electrostatic attraction induces nanocellulose swelling, substantially boosting nisin release [93]. Such a release mechanism achieves the controllable liberation of antimicrobial substances based on pH fluctuations in the packaging microenvironment. Marturano et al. [94] fabricated visible light-responsive polymeric nanocapsules. After 2 min of natural light irradiation, the release rate of coumarin-6 encapsulated in the nanocapsules reached 50%. Its core mechanism lies in precisely regulating the triggering wavelength of photosensitive materials to alter the internal chemical structure and physical properties, so as to accomplish the targeted and customized release of active carriers. Wu et al. [95] prepared composite aerogels encapsulating thymol with citrus pectin and cellulose nanofibers as substrates. The aerogels possess outstanding hygroscopicity and sustained-release capacity. They rapidly absorb ambient moisture to induce partial structural destruction of the packaging materials and release loaded thymol, presenting favorable antimicrobial activity against *S. aureus*. Lin et al. [96] constructed a dual-responsive intelligent controlled-release system toward β-glucuronidase and serine protease. The system was obtained by embedding cinnamaldehyde-loaded hyaluronic acid/carboxymethyl chitosan nanoparticles into silk fibroin nanofibers through electrospinning. In beef storage tests, this system significantly inhibited the growth and reproduction of *E. coli* and *S. typhimurium* after 5 days of storage at 4 °C and 25 °C. It is worth noting, nevertheless, that the above studies are not all based on aquatic products: the nanocellulose-nisin hydrogel combined with the visible-light-responsive nanocapsules were characterized for their release kinetics using a model compound [92], the thymol-loaded aerogel was tested in vitro against *S. aureus* [93], and the dual-responsive cinnamaldehyde system was validated in beef rather than in fish or shellfish [94]. For the temperature-sensitive polyurethane film [91], the food matrix is not specified. Given the characteristics of aquatic products, the responsiveness and release behaviour of these systems must be re-examined in aquatic matrices before their advantage can be claimed.

Notably, aquatic products are nutrient-dense but present potential allergenic risks. Suitable processing strategies, including high-pressure treatment, have been proven to effectively mitigate allergenicity [97,98]. Hence, combining hurdle-based antibacterial preservation with hypoallergenic processing is a promising research avenue. The following gaps require further study.

(i) Mechanisms of interaction among hurdle factors. Presently, most hurdle-technology-related research is limited to macroscopic assessment of preservation performance, with insufficient insight into microbial metabolism, bacterial interactions, and molecular inhibition mechanisms under multi-hurdle synergy.

(ii) Predictive models. Hurdle formula optimization largely depends on experimental screening, without quantitative models and standardized parameters derived from the growth kinetics of specific spoilage organisms. Computer-driven kinetic predictive models can drastically cut down unproductive experiments and enhance the reliability of hurdle combinations.

(iii) Behaviour of mixed microbial communities. High-throughput 16S rRNA sequencing, proteomics, and multi-omics tools (metagenomics, metabolomics) should be employed to unravel how combined hurdles regulate community succession, gene expression, and quorum sensing. This multi-omics framework enables characterization of shifts in microbial community succession, in situ gene expression and quorum-sensing signaling. Subsequent targeted assays, including QS reporter strains, LC-MS/MS quantification and mutant/complementation experiments, can further validate these molecular findings.

(iv) Validation in real aquatic food matrices. Moreover, targeted hurdle systems tailored for spoilage microbiota of different aquatic foods to realize precise alignment of antibacterial sites and spoilage-related metabolic pathways deserve further exploration. Validation of the active compounds partitioning into proteins and lipids and the spoilage consortium differs under industrially relevant conditions.

## 6. Conclusions

This paper systematically addresses the common spoilage organisms reported in aquatic products. In particular, SSOs including *Shewanella* spp. and *Pseudomonas* spp. are key contributors to aquatic product spoilage, and the effective control of such microorganisms represents the core challenge in aquatic product preservation. As a promising preservation strategy, hurdle technology integrates a variety of physical, chemical, and biological preservation factors. Its antimicrobial and fresh-keeping effects are achieved through multiple pathways. Combined hurdle technology inhibits the metabolism and proliferation of spoilage microbes at multiple targets through synergistic interactions among various factors, achieving superior preservation performance and emerging as a prevailing research hotspot for aquatic product preservation. Furthermore, this review provides an in-depth illustration of the synergistic preservation mechanism and industrial application potential of combined hurdle technology. It also summarizes aquatic preservation strategies aimed at suppressing typical spoilage organisms and introduces emerging techniques, such as intelligent controlled-release antibacterial packaging. It is expected to provide theoretical support for the iterative development of novel long-acting preservation technologies for aquatic products.

## Figures and Tables

**Table 1:** Comparison of common hurdle technologies.

Types	Technologies	Antimicrobial Mechanisms	Spoilage Organisms	Advantages
Physical approaches	Low-temperature preservation [56]	Suppresses microbial proliferation Inhibits enzymatic activity	*Vibro parahaemolyticus*	Easy to operate Well preserves the original flavor
	MAP [15]	Inhibits microbial proliferation Inhibits lipid oxidation	Enterobacteriaceae	High efficiency Maximally preserving the original color
	Ozone preservation [37]	Alter cell membrane permeability Suppress microbial metabolism	*P. fluorescens* yeast and lactic acid bacteria	Non-toxic Harmless Residue-free
	Ultrasonic [4]	Cavitation effects eliminate microorganisms Inactivate endogenous enzymes Induce protein denaturation	*P. aeruginosa* *Streptococcus mutans* *Staphylococcus epidermidis*	Less loss of nutrients Pollution-free Residue-free
	Irradiation preservation [57,58]	Destroy microbial DNA Alter cell membrane permeability	*Pseudomonas* *B. thermosphacta* *Lactobacillus* psychrophilic bacteria and H_2_S-producing bacteria	Safe Eco-friendly Efficient Easy to operate
	Low-temperature plasma [68,69]	Disrupt the bacterial structure Induce the leakage of intracellular components	*P. aeruginosa* *V. parahaemolyticus*	Efficient sterilization Low-temperature Residue-free, eco-friendly
	Photodynamic inactivation [70,71]	Disrupt microbial cellular structure Promote lipid oxidation	*Acinetobacter baumannii* *P. fluorescens*	Efficient at low temperatures Residue-free Less likely to induce drug resistance
Chemical approaches	Butylated hydroxytoluene, butylated hydroxyanisole, and other chemical preservatives [59]	Suppresses enzyme-related systems Induces DNA mutations Leads to cell-wall damage	*Bacillus subtilis* *E. coli* *S. aureus*	Good antibacterial effect Broad spectrum Low cost
Biological approaches	Antimicrobial biopreservatives such as nisin and chitosan [6,62]	Inactivate or suppress microbial proliferation Alter the structure and permeability of the cell membrane	*P. aeruginosa* *Psychrotrophic pseudomonas*	Safe Non-toxic Excellent biodegradability
	Lysozyme and other enzymatic biopreservatives [3,63]	Disruption of structural integrity Breakage of the cell wall	Lysozyme has a significant antibacterial effect on Gram-positive bacteria, but is weak against Gram-negative bacteria	Low dosage Mild conditions, easy control Non-toxic profile

**Table 2:** Preservative effects of the combined hurdle technology.

Types	Approaches	Species of Aquatic Products	Spoilage Organisms	Efficacy
Physical–Physical approaches	Low-voltage electrostatic field combined with partial freezing [11]	Fish (chilled large yellow croaker)	*Pseudomonas* *Psychrobacter*	Extended the shelf life by at least 6 days
	Ultra-high pressure combined with magnetic field [10]	Fish (grass carp fillets)	*Pseudomonas* *Aeromonas* *Shewanella* *Carnobacterium* *Lactococcus*	Extended the shelf-life by at least 10 days
Physical–Chemical approaches	Acidic electrolyzed water ice [31]	Shrimp (*L. vannamei*)	*Psychrobacter* *Shewanella* *Flavobacterium*	A strong antibacterial rate was still maintained on day 7
	Fibers coating combined with refrigeration [72]	Fish (salmon)	*P. fluorescens* P07	Muscle-tissue integrity of salmon slices persisted for 192 h during storage
Physical–Biological approaches	Low temperature combined with plant polyphenols [73]	Fish (channel catfish fillets)	*Proteobacteria*	Acceptable physicochemical quality was preserved in channel catfish fillets after 11 days of refrigerated storage
	Tea polyphenols, salinity, and pH [18]	Fish (large yellow croaker)	*Proteus vulgaris* *Hafnia alvei*	Microbial growth was fully suppressed under the optimized conditions
	Low temperature combined with bacteriocins [75]	Fish (fresh sardines)	*Pseudomonas* spp. *Vibrio* spp. *S. aureus* Enterobacterales Coliforms	Ensured high microbial safety of fresh sardines during 12 days of storage
	Low temperature combined with lysozyme [76]	Fish (*L. crocea*)	*Pseudomonas* spp.	Sensory scores of large yellow croaker samples remained acceptable over 35-day storage
Biological–Chemical approaches	Polysaccharide-based composite coatings incorporated with gallic acid [7]	Fish (*T. trachurus* fillets)	*Pseudomonas* spp. Enterobacteriaceae H_2_S-producing microorganisms and lactic acid bacteria	Preserve the color and texture of fish fillets during the 13-day refrigerated storage
	Protein-based composite coatings incorporated with plant essential oils [77]	Fish (rainbow trout)	*Pseudomonas* spp. Enterobacteriaceae, mesophilic bacteria, and psychrotrophic bacteria	Over a 12-day storage period, the treatment group maintains desirable sensory properties covering odor and color
Bio–Biological approaches	Chitosan combined with ε-polylysine [81]	Shrimp (*Fenneropenaeus chinensis*)	*	Increased the shelf-life by 4–6 days following this treatment

* Means no spoilage organisms were assayed in the original reference.

## Data Availability

No new data were created or analyzed in this study. Data sharing is not applicable to this article.

## Abbreviations

The following abbreviations are used in this manuscript:

MAP	modified atmosphere packaging
QS	quorum sensing
ROS	reactive oxygen species
SSOs	specific spoilage organisms
TorA	trimethylamine-N-oxide reductase
TVB-N	total volatile basic nitrogen
VP	vacuum packaging
